# Letter to the editor: Japan's emergency approval system under the COVID‐19 pandemic

**DOI:** 10.1002/hsr2.1770

**Published:** 2023-12-18

**Authors:** Yudai Kaneda, Akihiko Ozaki, Tetsuya Tanimoto

**Affiliations:** ^1^ School of Medicine Hokkaido University Hokkaido Japan; ^2^ Department of Breast and Thyroid Surgery Jyoban Hospital of Tokiwa Foundation Fukushima Japan; ^3^ Department of Internal Medicine Jyoban Hospital of Tokiwa Foundation Fukushima Japan

During the initial phase of the COVID‐19 pandemic, pharmaceutical companies in Japan could not develop novel drugs or vaccines of domestic origins. Drugs that had been approved overseas, such as Remdesivir (Veklury), were imported with special approval. However, the only route available for drugs developed in Japan and with no overseas approval was the normal approval process, which took a long time to get through. To encourage domestic production, in May 2022, Japan's Pharmaceuticals and Medical Devices Law was revised, and an emergency approval system was newly implemented, which resembles long‐criticized Japan's conditional approval system of regenerative medicine.^1^ Namely, it has lowered the requirements of regulatory approval for newly developed and non‐replaceable agents that can be estimated to be possibly safe and effective based on preliminary clinical trials in case of an emergency such as a pandemic. The Ministry of Health, Labor, and Welfare states that in case of the rapid increase of infected people, the difficulty in identifying the route of infection, and the strained medical products provision system as seen in the COVID‐19 pandemic, they will emergently approve a novel agent that has only limited clinical data to avoid the enormous impact on citizen's lives and the Japanese economy.^2^

The first candidate antiviral drug against mild‐to‐moderate COVID‐19 by a Japanese company, ensitrelvir fumaric acid (Xocoba), was submitted for approval in February 2022. When it was deliberated in July, because of statistically insignificant improvement in symptoms in phases IIa and IIb of a trial (T1221),^3^ emergency approval was postponed until the Phase III trial's results were available. As a result, although the original clinical trial protocol revised on July 8, 2022, had ‘time until recovery from 12 symptoms' as its primary endpoint, following a postponement of approval, by September 20, the endpoints were narrowed to five symptoms: “fatigue,” “fever,” “nasal discharge,” “sore throat,” and “cough,” with the assessment of more moderate to severe symptoms such as headache, chills, and myalgia being removed from the primary evaluation criteria. Consequently, the approval committee noted a significant difference in the recovery time for the main five symptoms,^4^ and the drug was granted emergency approval in November, becoming the first domestically produced oral COVID‐19 medication. However, when evaluating the initial 12 symptoms, no statistical significance was found in the recovery time, and the fact that the Japanese government had a contract with Shionogi for the production of doses for one million people before approval has fostered skepticism among experts regarding this authorization.^4^, ^5^

Specifically, in the revised study, it was reported that compared to the placebo group, the time to resolution of the five typical symptoms of the Omicron variant had been reduced by approximately 24 h in a statistically significantly shorter period.^4^ There was a 24.3‐h difference of 167.9 h in the drug group and 192.2 h in the placebo group; however, this is based on an integrated view of the results from all of Japan, Korea, and Vietnam, where clinical trials were conducted.^4^ In the subgroup analysis limited to Japanese patients, the difference was only 6.3 h, which did not reach statistical significance.^4^ Furthermore, the protocol prohibits the concomitant use of remedies other than acetaminophen; however, in actual clinical practice, cough suppressants and expectorants are usually used to treat COVID‐19‐infected patients. Therefore, some symptomatic improvement can be achieved with the combination of existing drugs, and the significance of a slight improvement in symptoms of mildly ill patients with the new drug would be diminished. Despite such concerns, the emergency authorization was granted for a domestically producible treatment targeting patients with a low risk of severe illness, as previously approved drugs like Pfizer's nirmatrelvir (Paxlovid) and Merck's molnupiravir (Lagevrio) remained limited to those at higher risk and to ensure the medication options amid the global instability and currency devaluation.^5^, ^6^

As such development illustrates, it is questionable whether the current Japanese regulatory policy for innovative medicine could contribute to citizens' health and welfare rather than the profits of domestic companies. The current case, reminiscent of the Japanese government's prioritization of domestically‐produced oral polio vaccines marketing until 2012, resulting in over 80 subsequent cases of vaccine‐associated paralytic poliomyelitis even after the 1981 eradication of the wild poliovirus, underscores a prevailing adherence to traditional infallibility at the detriment of patient welfare.^7^, ^8^ The Japanese government should prioritize evidence‐based regulatory science rather than capitalism‐based one, and only such attitudes would build credibility and contribute to medicine in Japan and beyond.

## AUTHOR CONTRIBUTIONS

**Yudai Kaneda**: Conceptualization; writing—original draft. **Akihiko Ozaki**: Writing—review and editing. **Tetsuya Tanimoto**: Writing—review and editing.

## CONFLICTS OF INTEREST STATEMENT

Dr Ozaki reported personal fees from Medical Network Systems Inc. and Kyowa Kirin co. ltd. outside the submitted work. Dr Tanimoto reported personal fees from Medical Network Systems Inc. and Bionics co. ltd., outside the submitted work. The remaining author declares no conflict of interest.

## Data Availability

Data available on request from the authors
